# *LAG3* and Its Ligands Show Increased Expression in High-Risk Uveal Melanoma

**DOI:** 10.3390/cancers13174445

**Published:** 2021-09-03

**Authors:** Zahra Souri, Annemijn P. A. Wierenga, Wilma G. M. Kroes, Pieter A. van der Velden, Robert M. Verdijk, Michael Eikmans, Gregorius P. M. Luyten, Martine J. Jager

**Affiliations:** 1 Department of Ophthalmology, Leiden University Medical Center, 2300 RC Leiden, The Netherlands; z.souri@lumc.nl (Z.S.); a.p.a.wierenga@lumc.nl (A.P.A.W.); p.a.van_der_velden@lumc.nl (P.A.v.d.V.); g.p.m.luyten@lumc.nl (G.P.M.L.); 2 Department of Clinical Genetics, Leiden University Medical Center, 2300 RC Leiden, The Netherlands; w.g.m.kroes@lumc.nl; 3 Department of Pathology, Leiden University Medical Center, 2333 ZA Leiden, The Netherlands; r.m.verdijk@lumc.nl; 4 Department of Pathology, Section Ophthalmic Pathology, Erasmus Medical Center, 3015 GD Rotterdam, The Netherlands; 5 Department of Immunology, Leiden University Medical Center, 2333 ZA Leiden, The Netherlands; m.eikmans@lumc.nl

**Keywords:** uveal melanoma, inflammation, metastasis, immunotherapy, LAG3, Galectin-3

## Abstract

**Simple Summary:**

Uveal melanoma (UM) is a rare type of intraocular malignancy, which often gives rise to metastases. While treatment with immune checkpoint inhibitors is often effective in the treatment of cutaneous melanoma metastases, it is hardly effective in the case of UM metastases. Lymphocyte-activation gene 3 (LAG3) is a recently recognized immune checkpoint; we determined the distribution of *LAG3* expression and its ligands in three sets of primary UM. High-risk UM (epithelioid cell type, loss of chromosome 3/BAP1 staining) had a higher expression of LAG3 and its ligands, which correlated with the presence of infiltrating immune cells. We concluded that not only *LAG3* but also its ligands *Galectin-3* and *HLA class II* are especially expressed in high-risk UM and may be a target for adjuvant immunotherapy in UM.

**Abstract:**

Uveal melanoma (UM) is a rare ocular malignancy which originates in the uveal tract, and often gives rise to metastases. Potential targets for immune checkpoint inhibition are lymphocyte-activation gene 3 (LAG3) and its ligands. We set out to analyse the distribution of these molecules in UM. The expression of mRNA was determined using an Illumina array in 64 primary UM from Leiden. The T lymphocyte fraction was determined by digital droplet PCR. In a second cohort of 15 cases from Leiden, mRNA expression was studied by Fluidigm qPCR, while a third cohort consisted of 80 UM from TCGA. In the first Leiden cohort, *LAG3* expression was associated with the presence of epithelioid cells (*p* = 0.002), monosomy of chromosome 3 (*p* = 0.004), and loss of BAP1 staining (*p* = 0.001). In this Leiden cohort as well as in the TCGA cohort, *LAG3* expression correlated positively with the expression of its ligands: *LSECtin*, *Galectin-3*, and the HLA class II molecules *HLA-DR*, *HLA-DQ*, and *HLA-DP* (all *p* < 0.001). Furthermore, ligands *Galectin-3* and *HLA class II* were increased in monosomy 3 tumours and the expression of *LAG3* correlated with the presence of an inflammatory phenotype (T cell fraction, macrophages, *HLA-A* and *HLA-B* expression: all *p* < 0.001). High expression levels of *LAG3* (*p* = 0.01), *Galectin-3* (*p* = 0.001), *HLA-DRA1* (*p* = 0.002), *HLA-DQA1* (*p* = 0.04), *HLA-DQB2* (*p* = 0.03), and *HLA-DPA1* (*p* = 0.007) were associated with bad survival. We conclude that expression of the LAG ligands *Galectin-3* and *HLA class II* strongly correlates with *LAG3* expression and all are increased in UM with Monosomy 3/BAP1 loss. The distribution suggests a potential benefit of monoclonal antibodies against LAG3 or Galectin-3 as adjuvant treatment in patients with high-risk UM.

## 1. Introduction

Uveal melanoma (UM) is a rare ocular malignancy which is especially seen in people with a fair skin and light eyes [1,2]. Up to 50% of patients develop metastases and no improvement in survival has occurred over the last 50 years [3,4]. The presence of an inflammatory phenotype is associated with a bad prognosis and involves the presence of a mixed leukocytic infiltrate, which is made up of tumour-infiltrating lymphocytes (TIL), tumour-associated macrophages (TAM), and an increased HLA class I and II expression [5,6,7,8,9]. In spite of the presence of large numbers of lymphocytes and macrophages, local immune responses are not effective against intraocular tumours and do not inhibit their growth and metastases formation; instead, the presence of infiltrating leukocytes seems to stimulate growth [10,11,12]. Overall, one can regard UM as having an immunosuppressive environment, which is characterized by the presence of FOXP3+ regulatory T cells [8], while macrophages may function as myeloid-derived suppressor cells [13]. We have suggested that genetic changes, specifically loss of BAP1 expression, were responsible for the influx of macrophages and lymphocytes, as well as for creating the immunosuppressive environment [14]. In agreement with this, another study also showed the association between loss of BAP1 and the infiltration of leukocytes and upregulation of genes related to immunosuppression using CYTOF technology and, furthermore, showed that such genes are expressed in UM metastases [15].

In order to obtain an effective immune response, it is important to find the reason behind the unresponsiveness of the immune cell population. T cell responses are modulated through binding of stimulatory and inhibitory ligands to cell surface receptors. Some of these ligands are known as immune checkpoints, which can prevent immune overstimulation and auto-immune responses. Well-known immune checkpoints are cytotoxic T-lymphocyte-associated protein 4 (CTLA-4) and programmed cell death protein-1 (PD-1) [16,17]. Another immune checkpoint is lymphocyte-activation gene-3 (LAG3), which is present on the surface of T cells, NK cells, and plasmacytoid dendritic cells [18]. The LAG3 protein forms a stable connection with HLA class II via its 30-amino acid loop structure, and selectively binds to peptide-containing major histocompatibility class II (MHCII) molecules [19,20]. Under normal circumstances, LAG3 may help to prevent auto-immune responses, or excessive responses against viral infections [21]. However, tumour cells may use immune checkpoints to avoid immune recognition and exhaust cytotoxic T cells. LAG3 is highly associated and synergistic with PD-1 as it is co-expressed with this immune checkpoint on CD4 and CD8 T cells: one study found that more macrophages, CD3, CD4, and CD8 T cells were present in murine tumours when LAG3 and PD-1 were both knocked out, which suggests that the combined deletion of these two factors alters regulator T cell homeostasis while enhancing tumour immunity [22].

Recently, Durante et al. called attention to the potential role of LAG3 in UM, indicating that LAG3 may be the dominant exhaustion marker in this malignancy [23]. Single cell RNA sequencing showed that most CD8 cytotoxic T cells in UM expressed LAG3 at a high level. The authors suggested that this was the reason that anti-PD-1 and anti-CTLA-4 therapies were not effective [24,25]. Figueiredo et al. independently reported on the presence of LAG3 in UM, and using the TCGA data, showed that an increased expression is related to loss of *BAP1* [15]. They reported an association between a high *LAG3* expression and a high rate of metastases.

Several studies have addressed the beneficial usage of anti-*LAG3* therapy in different malignancies: one monoclonal anti-LAG3 antibody, IMP321, was able to activate antigen-presenting cells (APCs) and T cells in breast cancer [26]. Another anti-LAG3 therapeutic approach proved efficacious with a high safety profile when it was combined with anti-PD-1 blockade in cutaneous melanoma patients [27,28]. 

While LAG3 is expressed on regulator T cells, it may interact with multiple cell surface receptors, such as LSECtin, Galectin-3, and HLA class II. In general, the effectiveness of targeted therapies is influenced by the expression of the target, and this also applies to the use of monoclonal antibodies against immune checkpoints; with this assumption, we analysed not only the expression of *LAG3* but also of its ligands in two new sets of primary UM as well as in the TCGA dataset to determine how to identify patients who can be targeted for adjuvant therapy.

## 2. Materials and Methods

### 2.1. Study Population

UM tissue was obtained from two groups of patients from the Leiden University Medical Center (LUMC) in Leiden, The Netherlands: the first group consisted of 64 patients who underwent an enucleation for UM between 1999 and 2008, of which 51% were male and 49% were female. The mean age at the time of enucleation was 61 years. The mean follow-up time (defined as the time period between enucleation and death) was 83 months (range 2 to 229 months). Follow-up was updated in 2020. At the end of follow up, 17 (27%) patients were alive, 37 (58%) patients had died because of metastasis, four (6%) had died because of other causes and six (9%) died with the cause of death unknown; the second group from Leiden was made up of 15 patients who underwent an enucleation for UM at the LUMC between 2016 and 2017: 60% of these patients were male while 40% were female. The mean age at the time of enucleation was 59 years; the mean follow-up time was 26 months (range 9 to 34 months). At the end of follow up, 13 (87%) patients were alive, while 2 (13%) patients had died because of metastases. We also looked at mRNA levels of tumours included in the TCGA database (*n* = 80) [9].

### 2.2. Chromosome Status

DNA was isolated from archived frozen tumour material using the QIAmp DNA Mini kit (Qiagen, Venlo, The Netherlands). Chromosome 3 aberrations were detected by SNP analysis using the Affymetrix 250K_NSP and Affymetrix SNP 6.0 array [14,29].

### 2.3. Illumina Array

Gene expression was determined on RNA obtained from archived frozen tumour material using the Illumina HT12v4 array (Illumina, Inc., San Diego, CA, US) for *LAG3*, *Galectin-3*, *LSECtin*, *HLA class II* (*DRalpha, DQalpha1, DQbeta2, DPalpha1*), and immune cell markers (*CD3, CD4, CD8, CD68, CD163*), as described previously [30].

### 2.4. T Lymphocyte Fraction

The T cell fraction was obtained as previously described, by using a digital droplet PCR (ddPCR) assay directed at a specific locus of the *TCR-β* gene [12,31].

### 2.5. Fluidigm qPCR

A Fluidigm qPCR (Fluidigm Corporation, South San Francisco, CA, USA) was performed on 15 UM tumours from Leiden, as previously described [32]. Briefly, total RNA (50–200 ng) was used for cDNA synthesis. Complementary DNA was diluted ten times (1.25 µL) and amplified with 2.5 µL of Taqman Preamp master mix (Applied Biosystems, Foster City, CA, USA) and 1.25 µL pooled primer mix for 14 cycles. QPCR reactions were performed using Eva-green dye and the final results were collected using the BioMark HD system (Fluidigm). Glyceraldehyde 3-phosphate dehydrogenase (*GAPDH*), β-actin, hypoxanthine phosphoribosyltransferase 1 (*HPRT-1*), ribosomal protein L13a (*RPL13a*) and hydroxymethylbilane synthase (*HMBS*) were used as reference genes. The geometric mean of these reference genes was used to standardize the gene expression signals of interest. The normalized data were log-transformed using Z-scores.

### 2.6. Statistical Analysis

Data were analysed by SPSS version 22.0 (SPSS, nc., Chicago, IL, USA). Graphs were obtained by GraphPad Prism version 5.0 for windows (GraphPad Software, La Jolla, CA, USA). The Spearman correlation was performed for correlations between non-parametric data. The Mann–Whitney U test was used to compare non-normal groups. A log-rank test was used for the significance analysis of survival graphs.

## 3. Results

### 3.1. Association between LAG3 and High-Risk Characteristics of UM

**Figure 1:**
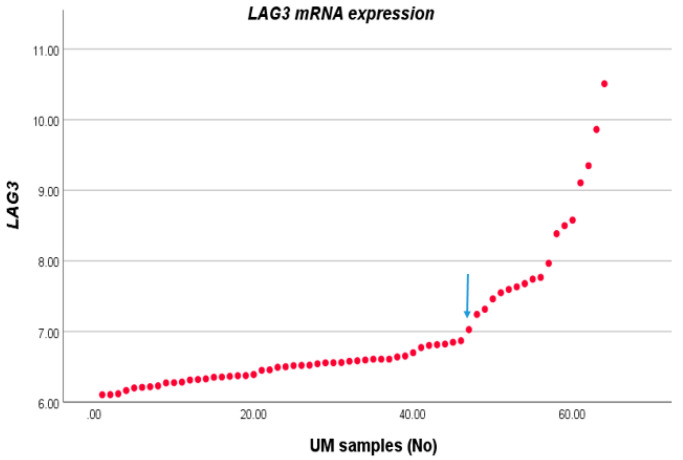
*LAG3* expression in UM. The *LAG3* expression level of 64 UM as seen in an Illumina array was sorted from low to high; we selected the inflection point of 6.87 to divide the cases into a low and high expression group.

We compared the expression level of *LAG3* with the distribution of clinico-pathological characteristics in a cohort of 64 cases of UM from Leiden, The Netherlands. For this, we first sorted *LAG3* expression from lowest to highest and observed two potential inflection points (Figure 1). We subsequently separated the cohort into two groups, with expression below and above the inflection point of 6.87. Forty-six tumours fell into the low expression group, and 18 into the high expression group. *LAG3* expression was increased in cases with age >60 years (*p* = 0.04), in the presence of epithelioid cells (*p* = 0.002), and loss of BAP1 staining as determined by immunohistochemistry (*p* = 0.001) (Table 1).

### 3.2. Association between LAG3 and Cell Surface Ligands

**Figure 2:**
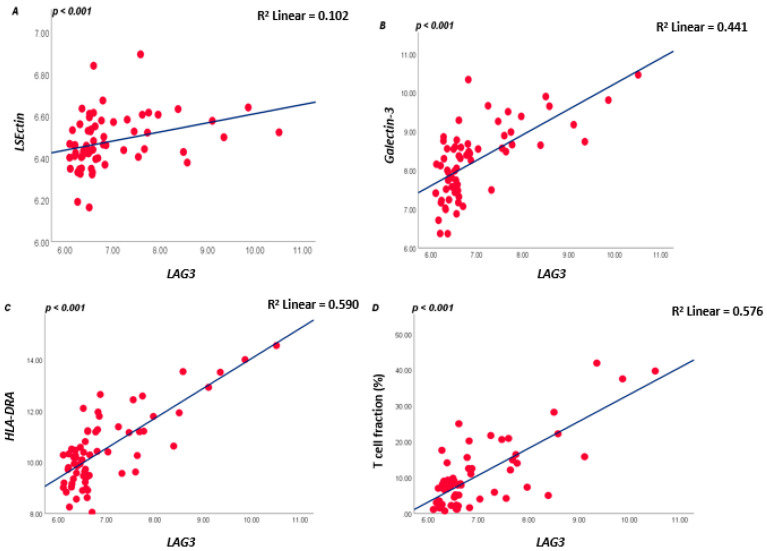
Correlation between *LAG3*, *LSECtin*, *Galectin-3*, and *HLA-DRalpha* in the 64 UM cohort from Leiden (**A**–**C**), and between *LAG3* and lymphocyte fraction as determined by ddPCR (**D**); a Spearman correlation was applied. *p* ≤ 0.05 is considered significant.

As LAG3 on T lymphocytes binds to ligands on APCs or tumour cells, we subsequently analysed which tumours express these ligands. LAG3 is known to form stable complexes with different cell surface receptors such as LSECtin, Galectin-3, and *HLA class II* isoforms. In the large Leiden cohort of 64 UM, expression of *LSECtin* and *Galectin-3* was positively correlated with *LAG3* (both *p* < 0.001). Messenger RNA expression levels of the *HLA class II* genes (*HLA-DRalpha*, *HLA-DQalpha*, and *HLA-DQbeta2* and *HLA-DPalpha1*) were positively associated with *LAG3* expression (all *p* ≤ 0.001) (Figure 2; Table S1A).

In order to validate our findings, we performed the same analyses using the TCGA database of 80 UM samples [9] and found almost identical results: *LAG3* expression showed a positive correlation with *LSECtin*, *Galectin-3*, *HLA-DRalpha*, *HLA-DQalpha*, *HLA-DQbeta2*, and *HLA-DPalpha* (all *p* ≤ 0.001) (Table S1B).

### 3.3. LAG3 and Inflammatory Phenotype

As we previously showed that monosomy of chromosome 3/loss of BAP1 are related to the presence of an inflammatory phenotype [7,14], we checked the associations of *LAG3* with immune cell markers: *LAG3* showed a positive association with the presence of TILs and TAMs (Spearman correlation coefficient, *CD3E* R = 0.727, *CD4* R = 0.596, *CD8A* R= 0.832, *CD68* R = 0.542, *CD163* R = 0.485, all *p* < 0.001), as well as with *HLA class I* expression (*HLA-A* probe 1 R= 0.712, *HLA-A* probe 2 R= 0.731, *HLA-B* R= 0.791, all *p* < 0.001) (Table S1A); the same pattern was observed in the TCGA cohort (Table S1B). 

As mRNA and RNAseq data show expression levels but not cell numbers, we additionally looked at the T-cell fraction as determined using a specifically developed ddPCR that quantifies the rearranged T-cell genes [12,31]. A good correlation was present between *LAG3* levels and the tumour’s T lymphocyte fraction (R = 0.553, *p* < 0.001, Figure 2D).

### 3.4. UM with Monosomy 3 Express Higher Levels of LAG3 Ligands Than with Disomy 3 

**Figure 3:**
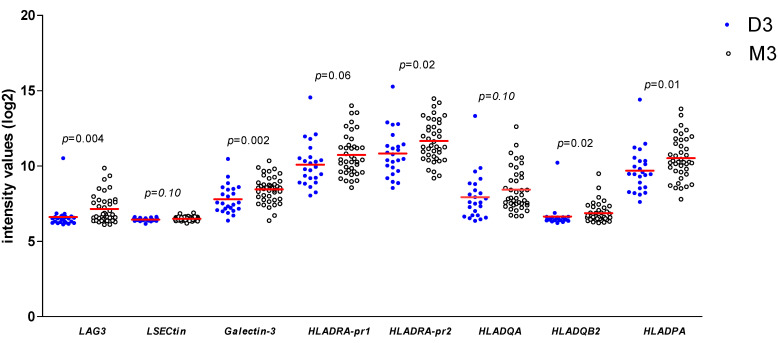
Comparison of gene expression of *LAG3*, *LSECtin*, *Galectin-3*, and several *HLA class II* molecules in 64 UM from Leiden between D3 (*n* = 24) and M3 (*n* = 40) UM; a Mann–Whitney U test was applied. Horizontal bars indicate mean gene expression. *p* ≤ 0.05 is considered significant.

As we found a positive association between *LAG3* expression and its ligands and confirmed the association between *LAG3* and loss of BAP1 expression/the presence of monosomy 3, we wondered whether expression of LAG3 ligands would similarly show associations with high-risk tumour characteristics. When looking at the Leiden 64-case cohort, expression of *Galectin-3* (*p* = 0.002), one of the probes for *HLA-DRalpha* (*p* = 0.02), *HLA-DQbeta2* (*p* = 0.02) and *HLA-DPalpha* (*p* = 0.01) was significantly higher in high-risk M3 tumours compared with low-risk D3 tumours (Figure 3).

In the TCGA cohort, we observed similar correlations: M3 tumours showed a higher expression than D3 tumours for *LAG3*, *LSECtin*, *Galectin-3*, *HLA-DRalpha*, *HLA-DQalpha*, and *HLA-DPalpha* (all *p* = 0.001, Mann–Whitney U test) (Figure S1).

As a control for the used mRNA/RNAseq expression levels, we analysed a second set of 15 UM from Leiden by Fluidigm qPCR, and compared the expression of *LAG3*, *HLA-DR*, and *Galectin-3* between D3 vs. M3 tumours (Table 2). Similar to prior results, the mean value of *LAG3* (*p* = 0.004) and *HLA-DR* (*p* = 0.004) was higher in M3 than in D3 tumours; this was not the case for *Galectin-3* (*p* = 0.61).

### 3.5. Association between LAG3 and Its Ligands with Survival

**Figure 4:**
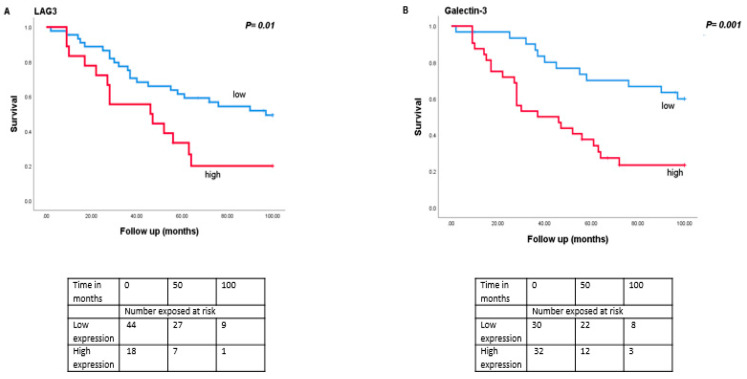

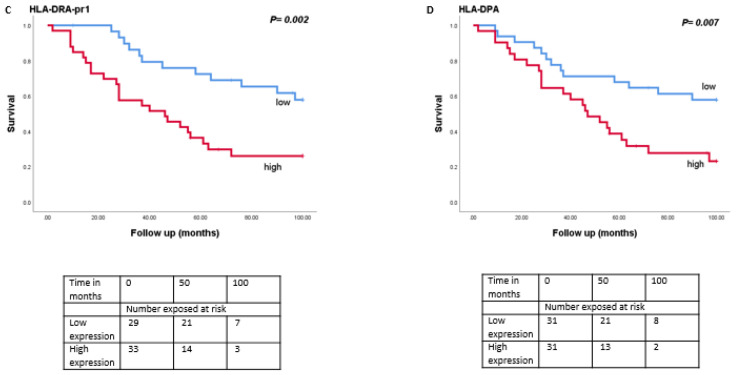
Kaplan-Meier survival curves based on mRNA expression of *LAG3*, *Galectin-3*, and *HLA class II* molecules. A log-rank test was used for statistical testing. *p* ≤ 0.05 is considered significant (**A**): *LAG3*, (**B**): *Galectin-3*, (**C**): *HLA-DR*-probe 1, (**D**): *HLA-DPA*.

As monosomy 3 is associated with a worse prognosis, we then analysed the relation between expression levels of *LAG3*, *LSECtin*, *Galectin-3*, and *HLA class II* molecules with survival in the Leiden set of 64 UM (Figure 4). Tumours with a high expression of *LAG3* (split at the inflection point into high and low, *p* = 0.01), *Galectin-3* (split at the median, *p* = 0.001), *HLA-DRalpha* (split at the median, *p* = 0.002), *HLA-DQalpha* (split at the median, *p* = 0.04), *HLA-DQbeta2* (split at the median, *p* = 0.02) and *HLA-DPalpha* (split at the median, *p* = 0.007) were associated with a significantly worse metastasis-related survival, while mRNA expression levels of *LSECtin* did not show an association with survival (*p* = 0.39).

When we split the cohort in D3 and M3 cases, no significant differences were observed in the survival of the D3 group (*n* = 24) (Figure S2) while in the M3 group (*n* = 40), increased levels of *Galectin-3* (split at the median, *p* = 0.05) and *HLA-DRalpha* (split at the median, *p* = 0.02) were significantly associated with a worse metastasis-related survival (Figure S3).

### 3.6. LAG3 and Immune Modulators

Combining several monoclonal antibodies directed against checkpoint inhibitors as treatment might boost the overall response to therapy in patients [27,28]. We therefore decided to look at the correlation between expression of *LAG3* and other immune modulators: *LAG3* showed a positive correlation with *PD-1* (two-tailed Spearman correlation coefficient R = 0.655, *p* < 0.001), *CTLA-4* (R = 0.298, *p* = 0.02), *IDO-1* (R = 0.759, *p* < 0.001) and one of the probes of *TIGIT* (R = 0.413, *p* = 0.001), while it did not show a significance association with *TIGIT*’s second probe (R = −0.207, *p* = 0.10) (Table S1A). Even higher associations were seen in the TCGA cohort (Table S1B).

## 4. Discussion

High-risk primary UM is associated with inflammation and its metastases are non-responsive to most current immunotherapy approaches. A treatment for metastases is eagerly awaited: many different studies have tested anti-PD-1 and anti-CTLA-4 therapies in UM patients, but results have been unsatisfactory, especially in comparison with metastasized cutaneous melanoma, with very few complete or partial responses [13,33,34]. As tumours with a high risk of developing metastases can be recognized because they carry specific chromosome aberrations and mutations, developing an early adjuvant therapy for preventing metastases would be most welcome. Such an adjuvant treatment proved useful in cutaneous melanoma patients [35]. Chromosome 3/BAP1 loss is a leading event in the development of the highest risk UM, which often give rise to metastases. A strong association was observed between chromosome 3/BAP1 loss and an inflammatory phenotype, consisting of a mixed arrangement of leukocytes and enhanced inflammation [7,8,14,15,36]. The recent study by Figueiredo et al. showed that BAP1 loss is associated with upregulation of multiple genes responsible for immunosuppression including *LAG3* [15]. 

The immune checkpoints that we specifically investigated were *LAG3* and its ligands. In our two Leiden cohorts, we had data on BAP1 immunohistochemistry, and we obtained long-term follow-up information. We confirmed that high levels of *LAG3* expression in UM were positively associated with high-risk tumour parameters, such as epithelioid/mixed cell type and chromosome 3/BAP1 loss, and described an association with the presence of inflammatory cells. We show in several datasets that not only a high expression of *LAG3* but also of its ligands is associated with bad survival rates. Recently, the group of Harbour commented that *LAG3* was the most dominant immune checkpoint molecule in UM [23]. They studied a small series of primary UM and metastases by single cell RNAseq analysis. It might be that the CD8 cytotoxic T cell population in UM is functionally exhausted by the LAG3-signaling cascade. Such a situation was described in follicular lymphoma, with T cells forming a heterogenic population: some of the T cells which expressed PD-1 were also LAG3 positive. The PD-1+LAG-3+ CD8 population was poor in cytokine production compared with the group which was LAG3 negative and hence was immunologically non-functional; moreover, in accordance with what we found in UM, the high level of *LAG3* correlated with worse survival in this disease [37]. Hoefsmit recently described that liver metastases from UM patients often contain LAG3-expressing lymphocytes [38].

One of the ligands for LAG3 is LSECtin, a member of the selectin family, which is highly expressed in liver tissue [39]. In one study, LSECtin was expressed on murine B16 melanoma cells: interaction between LSECtin and LAG3 led to inhibition of Interferon γ (IFNγ) secretion by *CD8* T cells and reduced their cytotoxic activity, promoting tumour growth [40]. We found positive associations of the expression of *LSECtin* with the expression of *LAG3* (*p* < 0.001), but no significant difference between D3 and M3 tumours.

Galectin-3 is a type of lectin with immune checkpoint inhibitory activity through its capacity to bind to LAG3 on CD8+ effector T cells. It can serve as a chemoattractant for macrophages and is reported to be highly expressed during inflammation [41]. One biological process in which this protein might be involved is the suppression of T cell-mediated lysis: Galectin-3 was shown to interact with LAG3 present on CD8 cells in pancreatic ductal adenocarcinoma. Depletion of *Galectin-3* improved CD8 cytotoxicity [42]. In cutaneous melanocytic lesions, melanoma and metastases more often displayed nuclear and cytoplasmic Galectin-3 than naevi [43]. In accordance with what we observed, the expression of *Galectin-3* was identified as a marker for a low overall survival of different cancers such as colorectal, ovarian, and non-small cell lung cancer, serving as an anti-apoptotic, proangiogenic and invasive agent [44]. It was reported that Galectin-3 promotes ocular inflammation and when knocked down, decreases angiogenesis signalling pathways in human endothelial cells [45]. From these studies and the association of the elevated expression of *Galectin-3* and *LAG3*, we can assume that the interaction between Galectin-3/LAG3 may take place in UM and may be a cause for the blunted activity of CD8 cells.

A third set of LAG3 ligands is made up of the HLA class II antigens, which are expressed on tumour cells, antigen-presenting cells, and a subset of T cells. These heterodimer cell surface molecules consist of alpha and beta chains. The HLA antigens can present antigens to CD4 T cells through binding to their T cell receptors. HLA class II molecules consist of three isotypes: DR, DQ, and DP which differ slightly in structure [46,47]. Cutaneous melanoma cells express high levels of HLA class II, which were shown to inhibit T cell anti-tumoural activity through their LAG3 receptor [48]. In UM, HLA class II is expressed in primary UM and carries prognostic significance [6]. In 1988, Jager et al. reported that HLA class II is expressed in UM, although less than in HLA class I. Our prior study showed that HLA-DQ was associated with increased numbers of infiltrating TILs [49]. When we compared expression levels between D3 and M3 tumours in the 64-case Leiden cohort, most class II molecules were higher in high-risk M3 tumours. We confirmed that *HLA class II* expression was enhanced in M3 tumours by analysing the TCGA data and by testing a second Dutch cohort of fifteen UM using Fluidigm PCR. It was been reported that HLA-DR is the dominant isoform for antigen-restricted T cell stimulation with HLA-DQ contributing less in different diseases [50,51,52].

IFNγ is a strong inducer for HLA class II molecules in UM [53]; we suggest a positive feedback loop between the tumour cells HLA class II expression, and the infiltrate: genetically bad tumours with loss of one chromosome 3 and a mutation in the BAP1 gene upregulate surface LAG3 ligands (LSECtin, Galectin-3, and HLA class II) to attract T cells which express LAG3. High-risk M3 tumours not only contain more infiltrating lymphocytes and macrophages, but also express higher levels of immune checkpoint inhibitors: we show that *LAG3* is co-expressed with other immune checkpoints (PD-1, *CTLA-4*, and *IDO-1*), suggesting that the combination of anti-checkpoint therapies might be more appropriate in UM than single anti-checkpoint antibodies, as others also find this appropriate in other diseases such as cutaneous melanoma [27].

## 5. Conclusions

Taken together, our data demonstrate elevated expression levels of not only *LAG3* but also of its ligands in high-risk M3 tumours. Moreover, we indicate that a positive association is found between the expression of *LAG3* and its ligands with other immune checkpoints and immune modulators. Experimental work in animals and clinical trials are indicated to validate the potential of using LAG3 and its ligands as targets for immunotherapy in UM.

## Figures and Tables

**Table 1:** *LAG3* expression compared with clinico-pathological and genetic characteristics in a set of 64 UM. Groups were separated according to the inflection point of 6.87; L: low expression, H: high expression. Using Pearson’s Chi-square test, *p* ≤ 0.05 is considered significant. Significant *p* values are indicated in bold. Numbers in brackets represent percentages.

	Number of Patients (%)		
Clinical and Histopathologic Characteristics			
	L	H	*p*
**Gender**			
Male	23 (36%)	10 (16%)	
Female	23 (36%)	8 (12%)	0.69
**Age (Years) at Enucleation (SD)**			
≤60	26 (41%)	5 (8%)	
>60	20 (31%)	13 (20%)	**0.04**
**Cell Type**			
Spindle	21 (33%)	1 (1%)	
Mixed/epithelioid	25 (37%)	17 (26%)	**0.002**
**Largest Tumour Diameter (LBD) in mm**			
<13.0 (median)	20 (31%)	7 (11%)	
≥13.0 (median)	26 (41%)	11 (17%)	0.74
**Tumour Prominence in mm**			
<8.0 (median)	24 (37%)	5 (8%)	
≥8.0 (median)	22 (34%)	13 (20%)	0.08
**Ciliary Body Involvement**			
Not involved	31 (48%)	9 (14%)	
Involved	15 (23%)	9 (14%)	0.2
**cTNM Stage** (*n* = 62)			
Stage I-IIB	27 (43%)	10 (16%)	
Stage IIIA-IIIB	17 (27%)	8 (13%)	0.67
**Metastasis**			
No	22 (34%)	4 (6%)	
Yes	24 (37%)	14 (22%)	0.06
**BAP1 status** (*n* = 55)			
BAP1 staining positive	24 (44%)	1 (2%)	
BAP1 staining negative	15 (27%)	15 (27%)	**0.001**

**Table 2:** Mean mRNA expression levels of different immune modulators defined by qPCR in UM tumours (*n* = 15). A Mann–Whitney U test was performed. *p* ≤ 0.05 is considered significant (indicated in bold).

Immune Modulator	D3 (*n* = 9)	M3 (*n* = 6)	
	Mean ± SD	Mean ± SD	*p*
***LAG3***	4 ± 3	98 ± 80	**0.004**
***Galectin-3***	1179 ± 1489	581 ± 345	0.61
***HLA-DR***	721 ± 606	4953 ± 4751	**0.004**

## Data Availability

Data from the Leiden cohort are partially accessible through GEO accession number GSE84976 (https://www.ncbi.nlm.nih.gov/geo/query/acc.cgi?acc=GSE84976, accessed date: 20 August 2021).

## Supplementary Materials

The following is available online at https://www.mdpi.com/article/10.3390/cancers13174445/s1, Figure S1: Comparison of gene expression of *LAG3*, *CIITA*, and *HLA Class II* in 80 UM from TCGA cohort (D3 (*n* = 37) vs. M3 (*n* = 43)), in relation to the tumor’s chromosome 3 status. A Mann–Whitney U test was applied. Horizontal bars indicate mean gene expression, Figure S2: Survival curves for *LAG3*, *Galectin-3*, *HLA-DRA* probe 1 and *HLA-DPA* in the Leiden D3 tumours (all split in two groups along the median), Figure S3: Survival curves for *LAG3*, *Galectin-3*, *HLA-DRA* probe1 and *HLA-DPA* in the Leiden M3 tumours (all split in two groups along the median), Table S1: Correlation between mRNA expression levels (determined by Illumina array) of *LSECtin*, *Galectin-3*, *HLA Class II* genes and infiltrate markers versus expression of *LAG3* in: A. the Leiden cohort (*n* = 64), and B. the TCGA cohort (*n* = 80). R = two-tailed Spearman correlation coefficient.

## References

[1] Metzelaar-Blok J.A., Ter Huurne J.A., Hurks H.M., Keunen J.E., Jager M.J., Gruis N. (2001). Characterization of melanocortin-1 receptor gene variants in uveal melanoma patients. Investig. Ophthalmol. Vis. Sci..

[2] Houtzagers L.E., Wierenga A.P.A., Ruys A.A.M., Luyten G.P.M., Jager M.J. (2020). Iris colour and the risk of developing Uveal Melanoma. Int. J. Mol. Sci..

[3] Kujala E., Mäkitie T., Kivelä T. (2003). Very long-term prognosis of patients with malignant uveal melanoma. Investig. Ophthalmol. Vis. Sci..

[4] Roelofsen C.D., Wierenga A.P., van Duinen S., Verdijk R.M., Bleeker J., Marinkovic M., Luyten G.P., Jager M.J. (2020). Five decades of enucleations for uveal melanoma in one center: More tumours with high risk factors, no improvement in survival over time. Ocul. Oncol. Pathol..

[5] Blom D.J., Luyten G.P., Mooy C., Kerkvliet S., Zwinderman A.H., Jager M.J. (1997). Human leukocyte antigen class I expression. Marker of poor prognosis in uveal melanoma. Investig. Ophthalmol. Vis. Sci..

[6] Ericsson C., Seregard S., Bartolazzi A., Levitskaya E., Ferrone S., Kiessling R., Larsson O. (2001). Association of HLA class I and class II antigen expression and mortality in uveal melanoma. Investig. Ophthalmol. Vis. Sci..

[7] Maat W., Ly L.V., Jordanova E.S., De Wolff-Rouendaal D., Schalij-Delfos N.E., Jager M.J. (2008). Monosomy of chromosome 3 and an inflammatory phenotype occur together in uveal melanoma. Investig. Ophthalmol. Vis. Sci..

[8] Bronkhorst I.H.G., Vu T.H.K., Jordanova E.S., Luyten G.P.M., van der Burg S.H., Jager M.J. (2012). Different subsets of tumour-infiltrating lymphocytes correlate with macrophage influx and monosomy 3 in uveal melanoma. Investig. Ophthalmol. Vis. Sci..

[9] Robertson A.G., Shih J., Yau C., Gibb E.A., Oba J., Mungall K.L., Hess J.M., Uzunangelov V., Walter V., Danilova L. (2017). Integrative analysis identifies four molecular and clinical subsets in Uveal Melanoma. Cancer Cell.

[10] Niederkorn J.Y. (2009). Immune escape mechanisms of intraocular tumours. Prog. Retin. Eye Res..

[11] Ly L.V., Baghat A., Versluis M., Jordanova E.S., Luyten G.P.M., Van Rooijen N., van Hall T., Van Der Velden P.A., Jager M.J. (2010). In aged mice, outgrowth of intraocular melanoma depends on proangiogenic M2-type macrophages. J. Immunol..

[12] De Lange M.J., Nell R., Lalai R.N., Versluis M., Jordanova E.S., Luyten G.P., Jager M.J., Van Der Burg S.H., Zoutman W.H., van Hall T. (2018). Digital PCR-Based T-cell Quantification-Assisted deconvolution of the microenvironment reveals that activated macrophages drive tumour inflammation in Uveal Melanoma. Mol. Cancer Res..

[13] Jager M.J., Shields C.L., Cebulla C.M., Abdel-Rahman M.H., Grossniklaus H.E., Stern M.-H., Carvajal R.D., Belfort R.N., Jia R., Shields J.A. (2020). Uveal melanoma. Nat. Rev. Dis. Primers.

[14] Gezgin G., Dogrusöz M., Van Essen T.H., Kroes W.G.M., Luyten G.P.M., Van Der Velden P.A., Walter V., Verdijk R.M., van Hall T., Van Der Burg S.H. (2017). Genetic evolution of uveal melanoma guides the development of an inflammatory microenvironment. Cancer Immunol. Immunother..

[15] Figueiredo C.R., Kalirai H., Sacco J.J., Azevedo R.A., Duckworth A., Slupsky J.R., Coulson J.M., Coupland S.E. (2020). Loss of BAP1 expression is associated with an immunosuppressive microenvironment in uveal melanoma, with implications for immunotherapy development. J. Pathol..

[16] Ishida Y., Agata Y., Shibahara K., Honjo T. (1992). Induced expression of PD-1, a novel member of the immunoglobulin gene superfamily, upon programmed cell death. EMBO J..

[17] Wierenga A.P., Cao J., Luyten G.P., Jager M.J. (2019). Immune checkpoint inhibitors in uveal and conjunctival melanoma. Int. Ophthalmol. Clin..

[18] Triebel F., Jitsukawa S., Baixeras E., Roman-Roman S., Genevee C., Viegas-Pequignot E., Hercend T. (1990). LAG-3, a novel lymphocyte activation gene closely related to CD4. J. Exp. Med..

[19] Huard B., Mastrangeli R., Prigent P., Bruniquel D., Donini S., El-Tayar N., Triebel F. (1997). Characterization of the major histocompatibility complex class II binding site on LAG-3 protein. Proc. Natl. Acad. Sci. USA.

[20] Maruhashi T., Okazaki I.-M., Sugiura D., Takahashi S., Maeda T.K., Shimizu K., Okazaki T. (2018). LAG-3 inhibits the activation of CD4+ T cells that recognize stable pMHCII through its conformation-dependent recognition of pMHCII. Nat. Immunol..

[21] Andrews L.P., Marciscano A.E., Drake C.G., Vignali D.A.A. (2017). LAG3 (CD223) as a cancer immunotherapy target. Immunol. Rev..

[22] Woo S.-R., Turnis M.E., Goldberg M.V., Bankoti J., Selby M., Nirschl C., Bettini M.L., Gravano D.M., Vogel P., Liu C.L. (2012). Immune inhibitory molecules LAG-3 and PD-1 synergistically regulate T-cell function to promote tumoural immune escape. Cancer Res..

[23] Durante M.A., Rodriguez D.A., Kurtenbach S., Kuznetsov J.N., Sanchez M.I., Decatur C.L., Snyder H., Feun L.G., Livingstone A.S., Harbour J.W. (2020). Single-cell analysis reveals new evolutionary complexity in uveal melanoma. Nat. Commun..

[24] Ascierto P., Ferrucci P., Stephens R., Del Vecchio M., Atkinson V., Schmidt H., Schachter J., Queirolo P., Long G., Di Giacomo A. (2018). Phase II multicenter, single arm, open label study of nivolumab in combination with ipilimumab in untreated patients with metastatic uveal melanoma. Ann. Oncol..

[25] Rozeman E.A., Prevoo W., Meier M.A., Sikorska K., Van T.M., Van De Wiel B.A., Van Der Wal J.E., Mallo H.A., Grijpink-Ongering L.G., Broeks A. (2020). Phase Ib/II trial testing combined radiofrequency ablation and ipilimumab in uveal melanoma (SECIRA-UM). Melanoma Res..

[26] Duhoux F.P., Dirix L.Y., Huizing M.T. (2018). Combination of paclitaxel and a LAG-3 fusion protein (eftilagimod alpha), as a first-line chemoimmunotherapy in patients with metastatic breast carcinoma (MBC): Final results from the run-in phase of a placebo-controlled randomized phase II. J. Clin. Oncol..

[27] Ascierto P.A., Melero I., Bhatia S., Bono P., Sanborn R.E., Lipson E.J., Callahan M.K., Gajewski T., Gomez-Roca C.A., Hodi F.S. (2017). Initial efficacy of anti-lymphocyte activation gene-3 (anti–LAG-3; BMS-986016) in combination with nivolumab (nivo) in PTS with melanoma (MEL) previously treated with anti–PD-1/PD-L1 therapy. J. Clin. Oncol..

[28] Khan S., Carvajal R.D. (2021). Dual Immunological Checkpoint Blockade for Uveal Melanoma. J. Clin. Oncol..

[29] Versluis M., De Lange M.J., Van Pelt S.I., Ruivenkamp C.A.L., Kroes W.G.M., Cao J., Jager M.J., Luyten G.P.M., Van Der Velden P.A. (2015). Digital PCR Validates 8q Dosage as Prognostic Tool in Uveal Melanoma. PLoS ONE.

[30] Van Essen T.H., Van Pelt S.I., Bronkhorst I.H.G., Versluis M., Némati F., Laurent C., Luyten G.P.M., Van Hall T., Elsen P.J.V.D., Van Der Velden P.A. (2016). Upregulation of HLA Expression in Primary Uveal Melanoma by Infiltrating Leukocytes. PLoS ONE.

[31] Zoutman W.H., Nell R.J., Versluis M., van Steenderen D., Lalai R.N., Out-Luiting J.J., de Lange M.J., Vermeer M.H., Langerak A.W., van der Velden P.A. (2017). Accurate quantification of T cells by measuring loss of germline T-cell receptor loci with generic single duplex droplet digital PCR assays. J. Mol. Diagn..

[32] Wierenga A.P.A., Gezgin G., Van Beelen E., Eikmans M., Spruyt-Gerritse M., Brouwer N.J., Versluis M., Verdijk R.M., Van Duinen S.G., Marinkovic M. (2019). Soluble HLA in the aqueous humour of uveal melanoma is associated with unfavourable tumour characteristics. Cancers.

[33] Jochems A., Van Der Kooij M.K., Fiocco M., Schouwenburg M.G., Aarts M.J., Van Akkooi A.C., Berkmortel F.W.V.D., Blank C.U., Eertwegh A.J.V.D., Franken M.G. (2019). Metastatic uveal melanoma: Treatment strategies and survival—Results from the Dutch melanoma treatment registry. Cancers.

[34] Rodrigues M., De Koning L., Coupland S.E., Jochemsen A.G., Marais R., Stern M.-H., Valente A., Barnhill R., Cassoux N., Evans A. (2019). So Close, yet so Far: Discrepancies between Uveal and Other Melanomas. A Position Paper from UM Cure 2020. Cancers.

[35] Eggermont A.M., Sileni V.C., Grob J.-J., Dummer R., Wolchok J.D., Schmidt H., Hamid O., Robert C., Ascierto P.A., Richards J.M. (2016). Prolonged survival in Stage III melanoma with ipilimumab adjuvant therapy. N. Engl. J. Med..

[36] Souri Z., Wierenga A.P.A., Van Weeghel C., Van Der Velden P.A., Kroes W.G.M., Luyten G.P.M., Van Der Burg S.H., Jochemsen A.G., Jager M.J. (2019). Loss of BAP1 is associated with upregulation of the NFKB pathway and increased HLA class I expression in uveal melanoma. Cancers.

[37] Yang Z.-Z., Kim H.J., Villasboas J.C., Chen Y.-P., Price-Troska T., Jalali S., Wilson M., Novak A.J., Ansell S.M. (2017). Expression of LAG-3 defines exhaustion of intratumoural PD-1+ T cells and correlates with poor outcome in follicular lymphoma. Oncotarget.

[38] Hoefsmit E.P., Rozeman E.A., Van T.M., Dimitriadis P., Krijgsman O., Conway J.W., da Silva I.P., Van der Wal J.E., Ketelaars S.L.C., Bresser K. (2020). Comprehensive analysis of cutaneous and uveal melanoma liver metastases. J. Immunother. Cancer.

[39] Liu W., Tang L., Zhang G., Wei H., Cui Y., Guo L., Gou Z., Chen X., Jiang D., Zhu Y. (2004). Characterization of a novel C-type lectin-like gene, LSECtin: Demonstration of carbohydrate binding and expression in sinusoidal endothelial cells of liver and lymph node. J. Biol. Chem..

[40] Xu F., Liu J., Liu D., Liu B., Wang M., Hu Z., Du X., Tang L., He F. (2014). LSECtin expressed on melanoma cells promotes tumour progression by inhibiting antitumour T-cell responses. Cancer Res..

[41] Flotte T.J., Springer T.A., Thorbecke G.J. (1983). Dendritic cell and macrophage staining by monoclonal antibodies in tissue sections and epidermal sheets. Am. J. Pathol..

[42] Kouo T.S., Huang L., Pucsek A.B., Cao M., Solt S., Armstrong T.D., Jaffee E.M. (2015). Galectin-3 shapes antitumour immune responses by suppressing CD8+ T cells via LAG-3 and inhibiting expansion of plasmacytoid dendritic cells. Cancer Immune Res..

[43] Prieto V.G., Mourad-Zeidan A.A., Melnikova V., Johnson M.M., Lopez A., Diwan A.H., Lazar A., Shen S.S., Zhang P.S., Reed J.A. (2006). Galectin-3 expression is associated with tumour progression and pattern of sun exposure in melanoma. Clin. Cancer Res..

[44] Wang Y., Liu S., Tian Y., Wang Y., Zhang Q., Zhou X., Meng X., Song N. (2018). Prognostic role of galectin-3 expression in patients with solid tumours: A meta-analysis of 36 eligible studies. Cancer Cell Internat..

[45] Markowska A.I., Jefferies K.C., Panjwani N. (2011). Galectin-3 protein modulates cell surface expression and activation of vascular endothelial growth factor receptor 2 in human endothelial cells. J. Biol. Chem..

[46] Korman A.J., Boss J.M., Spies T., Sorrentino R., Okada K., Strominger J.L. (1985). Genetic Complexity and Expression of Human Class II Histocompatibility Antigens. Immunol. Rev..

[47] Trowsdale J., Young J.A.T., Kelly A.R., Austin P.J., Carson S., Meunter H., So A., Erlich H.A., Spielman R.S., Bodmer J. (1985). Structure, sequence and polymorphism in the HLA-D region. Immunol. Rev..

[48] Hemon P., Jean-Louis F., Ramgolam K., Brignone C., Viguier M., Bachelez H., Triebel F., Charron D., Aoudjit F., Al-Daccak R. (2011). MHC Class II Engagement by Its Ligand LAG-3 (CD223) Contributes to Melanoma Resistance to Apoptosis. J. Immunol..

[49] Jager M.J., van der Pol J.P., de Wolff-Rouendaal D., de Jong P.T., Ruiter D.J. (1988). Decreased expression of HLA class II antigens on human uveal melanoma cells after in vivo X-ray irradiation. Am. J. Ophthalmol..

[50] Karp D.R., Teletski C.L., Jaraquemada D., Maloy W.L., Coligan J.E., Long E.O. (1990). Structural requirements for pairing of alpha and beta chains in HLA-DR and HLA-DP molecules. J. Exp. Med..

[51] Ulvestad E., Williams K., Bø L., Trapp B., Antel J., Mørk S. (1994). HLA class II molecules (HLA-DR,-DP,-DQ) on cells in the human CNS studied in situ and in vitro. Immunology.

[52] Grifoni A., Moore E., Voic H., Sidney J., Phillips E., Jadi R., Mallal S., De Silva A.D., De Silva A.M., Peters B. (2019). Characterization of magnitude and antigen specificity of HLA-DP, DQ, and DRB3/4/5 restricted DENV-specific CD4+ T cell responses. Front. Immunol..

[53] De Waard-Siebinga I., Creyghton W.M., Kool J., Jager M.J. (1995). Effects of interferon alfa and gamma on human uveal melanoma cells in vitro. Br. J. Ophthalmol..

