# Supplementary material for: LAG3 and Its Ligands Show Increased Expression in High-Risk Uveal Melanoma
Source: Cancers (Basel). 2021 Sep 3;13(17):4445. doi: 10.3390/cancers13174445 (PMC8430821; doi:10.3390/cancers13174445)
Supplement: Supplementary file 1 [file cancers-13-04445-s001.zip › cancers-1345869-supplementary.pdf]

Supplementary Materials: *LAG3* and Its Ligands Show Increased Expression in High-Risk Uveal Melanoma

Zahra Souri, Annemijn P.A. Wierenga, Wilma G.M. Kroes, Pieter A. van der Velden, Robert M. Verdijk, Michael Eikmans, Gregorius P.M. Luyten and Martine J. Jager

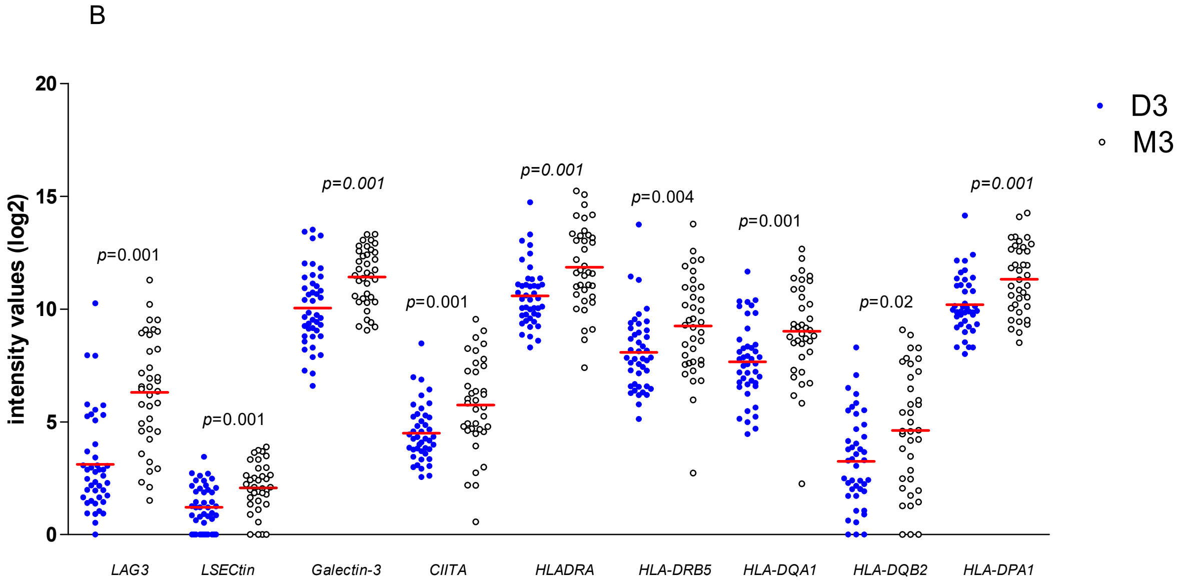

Figure S1. Comparison of gene expression of *LAG3*, *LSECtin*, *Galectin-3*, *CIITA*, and *HLA Class II* in 80 UM from TCGA cohort (D3 (n = 37) vs M3 (n = 43), in relation to the tumor's chromosome 3 status. A Mann-Whitney U test was applied. Horizontal bars indicate mean gene expression.

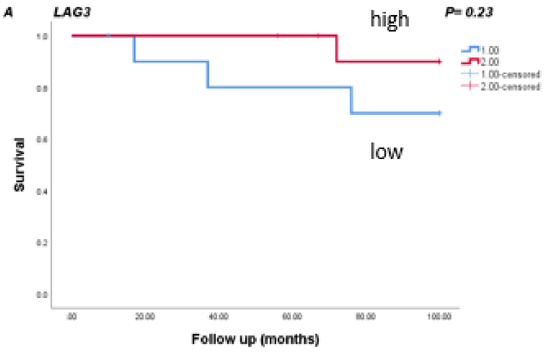

Time in months	0	50	100
	Number exposed at risk		
Low expression	10	8	3
High expression	12	11	4

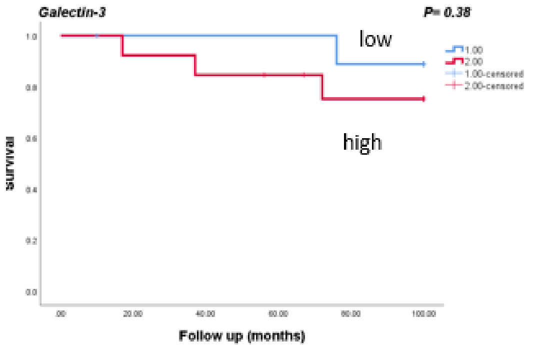

Time in months	0	50	100
	Number exposed at risk		
Low expression	9	9	4
High expression	13	10	4

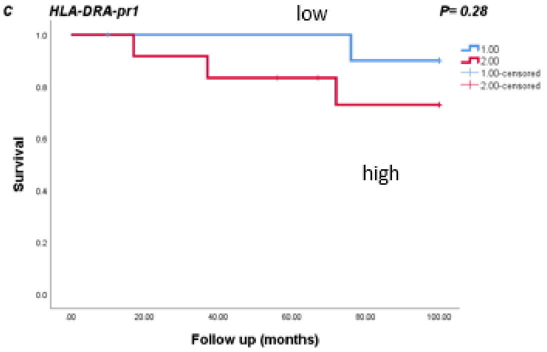

Time in months	0	50	100
	Number exposed at risk		
Low expression	10	10	4
High expression	12	9	3

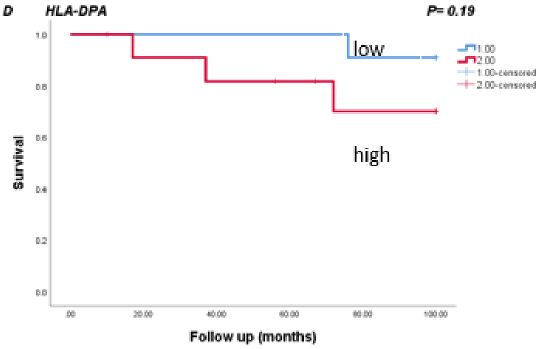

Time in months	0	50	100
	Number exposed at risk		
Low expression	11	11	5
High expression	11	8	3

Figure S2. Survival curves for *LAG3*, *Galectin-3*, *HLA-DRA*-probe 1 and *HLA-DPA* in the Leiden D3 tumours (all split into two groups along the median).

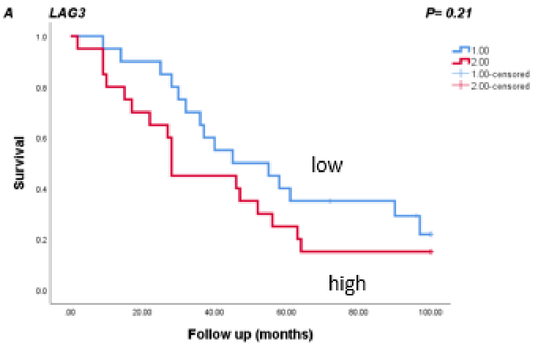

Time in months	0	50	100
	Number exposed at risk		
Low expression	20	9	1
High expression	20	7	1

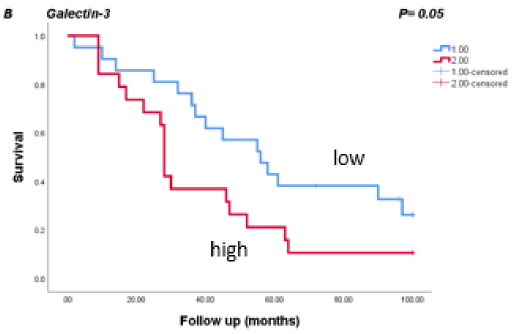

Time in months	0	50	100
	Number exposed at risk		
Low expression	21	11	2
High expression	19	5	1

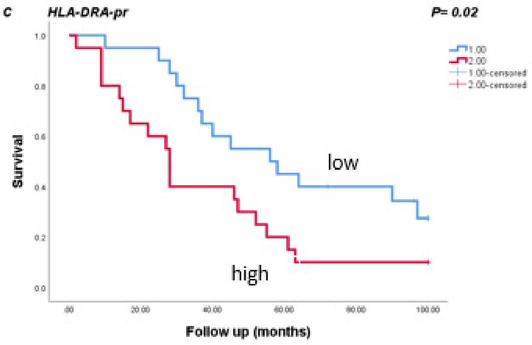

Time in months	0	50	100
	Number exposed at risk		
Low expression	20	10	2
High expression	20	6	1

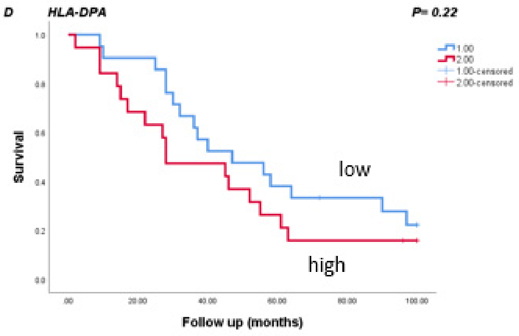

Time in months	0	50	100
	Number exposed at risk		
Low expression	21	9	2
High expression	19	6	1

Figure S3. Survival curves for *LAG3*, *Galectin-3*, *HLA-DRA*-probe 1 and *HLA-DPA* in the Leiden M3 tumours (all split into two groups along the median).

Table S1. Correlation between mRNA expression levels (determined by Illumina array) of *LSECtin*, *Galectin-3*, *HLA Class II* genes and infiltrate markers versus expression of *LAG3* in: A. the Leiden cohort (*n* = 64), and B. TCGA cohort (*n* = 80). R = two-tailed Spearman correlation coefficient. Pr stands for probe.

**A. Leiden cohort**	**LAG3**	
	**R**	***p***
LSECtin	.428	<0.001
Galectin-3	.672	<0.001
HLA-DRalpha-pr1	.633	<0.001
HLA-DRalpha-pr2	.669	<0.001
HLA-DQalpha1	.607	<0.001
HLA-DQbeta2	.735	<0.001
HLA-DPalpha1	.619	<0.001
CD3	.727	<0.001
CD4	.596	<0.001
CD8	.832	<0.001
CD68	.542	<0.001
CD163	.485	<0.001
HLA-A-pr1	.712	<0.001
HLA-A-pr2	.731	<0.001
HLA-B	.791	<0.001
PD1	.655	<0.001
CTLA-4	.298	0.02
IDO-1	.759	*<0.001*
TIGIT-pr1	.413	.001
TIGIT-pr2	-.207	.10

**B. TCGA cohort**	**LAG3**	
	**R**	***p***
LSECtin	.579	<0.001
Galectin-3	.666	<0.001
HLA-DR-alpha	.780	<0.001
HLA-DQalpha1	.787	<0.001
HLA-DQbeta2	.665	<0.001
HLA-DPalpha1	.805	<0.001
CD3	.886	<0.001
CD4	.609	<0.001
CD8	.846	<0.001
CD68	.301	.007
CD163	.638	<0.001
HLA-A	.773	<0.001
HLA-B	.796	<0.001
PD1	.878	<0.001
CTLA-4	.690	<0.001
IDO-1	.838	<0.001
TIGIT	.831	<0.001
